# How occupational physicians pay attention to the values of employees: a qualitative study

**DOI:** 10.1080/17482631.2024.2370069

**Published:** 2024-06-24

**Authors:** Pepijn R. Hagenaar, Willem van Rhenen, Arjan W. Braam, Lars van Tuin

**Affiliations:** a University of Humanistic Studies, Universiteit voor Humanistiek, Utrecht, The Netherlands; b Center for Strategy, Organization & Leadership, Nyenrode Business Universiteit, Breukelen, The Netherlands; c Department of Humanist Chaplaincy Studies for a Plural Society, University of Humanistic Studies, Utrecht, The Netherlands; d Department of Emergency Psychiatry and Department of Residency Training, Altrecht Mental Health Care, Utrecht, The Netherlands; e Social, Health and Organizational Psychology, Utrecht University, Utrecht, The Netherlands

**Keywords:** Health, well-being, values, intrinsic motivation, meaning making, occupational physician

## Abstract

**Purpose:**

The Dutch Association for Occupational Medicine considers employee values to be an essential pillar in occupational medicine. The occupational physician should focus on what an employee finds valuable. However, it is unclear how occupational physicians comply with this policy and pay attention to employee values. The present study aims to fill this gap by mapping to what extent occupational physicians pay attention to employee values.

**Method:**

We used an exploratory qualitative research method through in-depth interviews with 10 Dutch occupational physicians. Additionally, two non-participating observations were conducted.

**Results & conclusion:**

The results show that values remain mostly implicit and are applied intuitively or unconsciously but not explicitly. Hence, the ethical requirements of the Dutch Association for Occupational Medicine policy remain underexposed and under-executed. Multiple facets foster or impede a conversation about values. As far values were mentioned they were mainly extrinsic, social, and prestige-oriented. Intrinsic values were hardly mentioned. However, a few occupational physicians explicitly stated that they pay attention to values and reported that heeding to employee values contributes to better collaboration and decision-making with the employee. We argue that paying attention to intrinsic values may improve the overall work quality of occupational physicians and benefit employee well-being.

## Introduction

Values such as the Hippocratic oath form a crucial part of contemporary medicine (Askitopoulou & Vgontzas, 2018). However, values are subjective and include a sense of personal meaning-making regarding lifegoals, beliefs and convictions (Maslow, 1943; Sagiv et al., 2017; Schwartz, 2012). Additionally, people live their values mostly implicitly, whereas values transcend actions and deeds. Still, people tend to give priority to their most important values (Sagiv et al., 2017; Schwartz, 2012).

The policy guidelines of the Dutch Association of Occupational Medicine (NVAB) state that paying attention to employee values is one out of eight critical terms of the occupational physician’s mission: “We connect with what an employee finds valuable and what adds to meaningfulness in work” (NVAB, 2016, p. 2) (Appendix 3 describes the job and most important tasks of a Dutch occupational physician). The NVAB wants to underline the importance of meaning and value of health, e.g., as derived from Huber’s Positive Health dimensions (Huber et al., 2011). According to the description of the NVAB, personal values, relevant in life and work are at stake. This description draws on the capability approach, a theoretical framework for sustainable employability developed by Van der Klink et al. (2016). Sustainable employability in the context of the capability approach, refers to the level to which the work environment facilitates the possibility of living one’s values and motivation. Van der Klink et al. (2016) adopted the capability approach from Sen (2008): “a normative-empirical framework which asserts that human well-being should be primarily assessed from capabilities, or the real freedoms people have to be and do things they have reason to value” (Rijke et al., 2023, p. 402). Capabilities are the opportunities to achieve something and may also indicate whether one lives consistently with one’s values. The capability model aims to capture whether someone can utilize one’s own abilities and resources, and is being enabled by the environment (Sen, 2008).

Integrating sustainable employability and the capability approach in occupational medicine, requires occupational physicians to address employee work values during consultations. The occupational physician is supposed to examine what an employee finds important and which tasks are considered valuable or valueless (NVAB, 2016). Valueless tasks can be adjusted which would in turn, benefit the employee and the organization. More importantly, the experience of meaningful and valuable work correlates positively with health (less illness), productivity, pleasure at work, and job satisfaction (Steger, 2016; Steger et al., 2012). When the values of an organization are congruent with employee values, employee well-being tends to increase (Sagiv & Schwartz, 2000). Employees who experience their work as meaningful positively affect the organization because they are more motivated to take on (extra) tasks and roles and feel more connected to the organization (Breevaart et al., 2014; Steger, 2016).

Van der Klink et al. (2016) distinguishes seven values to measure sustainable employability, such as developing knowledge and skills, being involved in important decisions, or contributing to something valuable. The measure’s validity was established in an additional study (Abma et al., 2015), but we found no studies exploring and testing the practical application of the model. For the explorative nature of this study, we preferred a broader framework to deepen the possible outcomes. Since there are relatively few work value measures and even fewer with adequate reliability and validity (Rounds & Armstrong, 2021), we chose to rely on the values framework of Elizur et al. (1991). The authors distinguished 24 different work values also comprising the seven values from Van der Klink et al. (2016). These work values can be subdivided into four categories: intrinsic, extrinsic, prestige, and social work values (Jin & Rounds, 2012; Ros et al., 1999). Intrinsic work values motivate from within—it is a natural tendency to achieve something autonomously because it is interesting, pleasurable, explorative, or informative (Ryan & Deci, 2017). Here, the rewards of the activity lie in doing the activity itself (Reiss, 2012; Rosso et al., 2010; Ryan & Deci, 2017). Extrinsic work values refer to actions with a particular extrinsic incentive, such as profit-making (Ryan & Deci, 2017). Social and prestige values are also considered extrinsic (Baumeister, 1991; Twenge et al., 2010). Social work values refer to interpersonal meaning, such as “pleasant co-workers”, or a social meaning, such as “contributing to society.” Prestige work values relate to self-enrichment and superiority in comparison with others (Ros et al., 1999). Elizur et al. (1991) describe five prevalent values in a survey among Dutch employees: (1) job interest (intrinsic value), (2) achievement (prestige value), (3) co-workers (social value), (4) personal growth (intrinsic value) and (5) independence (prestige value). For the complete list, see Appendix 2.

Studies show that employee core work values (irrespective of the type of work values) are associated with meaningful work and meaning in life (Guillemin & Nicholas, 2022; Rosso et al., 2010). Employees who experience consistency between their personal (work) values and those of the organization tend to experience a feeling of meaningfulness (shared value systems) (Rosso et al., 2010). In particular, congruence with intrinsic values has been shown to offer greater life-satisfaction (Sheldon & Krieger, 2014), well-being (Unanue et al., 2017) and a greater success in goal attainment (Wrzesniewski et al., 2014).

Referencing the positive impact of including intrinsic values, the NVAB policy documents (2016) and the International Commission on Occupational Health (ICOH, 2014) advise that questions about important values belong to the main requirements in the work of an occupational physician. However, the policy documents and the sustainable employability approach do not describe *how* occupational physicians should pay attention to values in practice: it is a policy, not a practitioner’s instruction. Moreover, Plomp et al. (2012) found that many occupational physicians have difficulty complying with NVAB guidelines. They attribute this to the less familiarity with these guidelines, low perceived added value, and unclarity about the strictness in which they should be applied. Moreover, explicitly asking for, recognizing, and acknowledging values in conversations is difficult because values are usually lived implicitly and, therefore, remain hidden (Schwartz, 2012). Nevertheless, with decent reflection on one’s actions and listening with the heart one may identify important values (Geenen, 2017; Guillemin & Nicholas, 2022; Klamer, 2010; Mooren, 2011).

## Purpose and contribution

In the present study, we aim to examine whether and how occupational physicians pay (explicit) attention to the employee’s values. With this study, we add to the growing body of knowledge (Abma et al., 2016; Kuiper et al., 2016; Sen, 2008; Van der Klink et al., 2016) about the practical application of sustainable employability and the capability set. To date, little is known about how and to what extent occupational physicians pay attention to employee values in their conversations. Investigating the practical implications of values in conversations may provide insight into whether and how the NVAB guidelines are applied. Moreover, and in a practical sense, the present study informs occupational physicians about the role values play in conversations with employees and how addressing values may improve both the physician’s guidance and employee’s well-being.

## Research questions

To investigate this problem, we formulated three research questions:

1. What do occupational physicians perceive under the term “values” and do occupational physicians adhere to the NVAB guidelines?
2. Which factors foster and impede the attention to the employee’s values?
3. How do occupational physicians pay attention to the employee’s values and which work values do occupational physicians observe among employees?

## Method

### Research design

The present study was conducted at a national occupational health service company in the Netherlands. A qualitative approach was elaborated to investigate how occupational physicians pay attention to the employee’s values. Furthermore, we chose an explorative qualitative approach because no research has examined the motives, reasons, and interests of occupational physicians in the use of values in their practice.

### Data collection

We conducted semi-structured in-depth interviews to assess how occupational physicians pay attention to values, which values they find important, and which values they encounter during their conversations with the employees. In total, 10 interviews were held, each lasting approximately 45–75 minutes. The semi-structured interviews were conducted using an interview guide, in which ten main questions have been formulated. These ten questions were inferred from the theoretical framework and served as a first exploration of issues related to work values. Follow-up questions were employed to elaborate on the different topics and to arrive at more in-depth answers (Evers, 2015). Two example questions used in this study are: *To what extent are you concerned with employee values? How do you pay attention to values in conversations with employees?* The complete interview guide is included in Appendix 1. One researcher (P.H.) conducted the interviews. Before each interview the researcher introduced himself and told the participants about reasons and interest in the research topic. Conducting the interviews was somewhat hindered by the first protective measures against the spread of the COVID-19 virus. Therefore, it was not possible to conduct interviews during a live encounter. Instead, the interviews were conducted by telephone and via digital solutions. All interviews were audio recorded and field notes were made. This had the advantage that the interviewer could speak to occupational physicians who worked over the entire country. However, the non-verbal utterances were more difficult to register.

Prior to the data collection, the interviewer accompanied an occupational physician twice and attended several intakes with employees (just before the pandemic). These observations provided some further insight into how values play a role in occupational health practice. Two of these observations were used (with permission and informed consent) for this research. These were non-participating, unstructured observations. During the intake, the researcher sat next to the occupational physician while silently watching and taking notes on a small note block, so that the conversation was not, or minimally influenced in any way. Afterwards, these notes were processed into an ongoing narrative with thick description (Boeije, 2019) in which attention was paid to both verbal and non-verbal expressions, context, and subjective meanings.

### Recruiting participants

Using non-probability sampling (Boeije, 2019), we selected participants on the single criterion of whether they were an occupational physician. Therefore, no statistical representativeness has been achieved because the selection is not based on chance. The respondents were recruited via an invitation email that was sent to all 169 occupational physicians within an occupational health services company (with an equal male to female ratio). A total of 13 responded (8%), and 3 eventually dropped out (23%). After eight interviews the researchers noted saturation, at which no new information or insights emerged from the data. This ultimately resulted in ten interviews (77%). There were no interviews repeated. The respondents work in different sectors, such as industry, healthcare, or government. The invitation message included information about the researcher, reason for doing the research and explanation that the research pertained to the use of values during conversations with employees. However, an equal male to female ratio could not be realized, which resulted in seven male respondents and three female respondents. The average age of the respondents is relatively high (58.8 years, range from 46–65 years). Their average work experience was 28.8 years (range from 16–39 years).

### Data analysis

The interviews were transcribed, after which they were shared with the respondent for approval before adding them to the analysis. The two additional observations were written out using thick description (Boeije, 2019). The transcripts and extensive descriptions were thematically analysed with ATLAS.ti 8. Because of the explorative nature of this study and the lack of empirical knowledge, there is little known about how occupational pay attention to employee values. Therefore, the first step consisted of inductive coding. This coding was used to get a first impression of the data and to discover some relevant findings. Then, theoretical deductive codes were created, based on the 24 work values from Elizur et al. (1991) and main categories intrinsic, extrinsic, social, and prestige-oriented values (Ros et al., 1999) (see Appendix 2). These theoretical deductive codes were compared with the data, which together with the inductive codes yielded relevant quotations that were suitable to answer the research questions. These quotations covered and illustrated the most important themes in the interviews. The most frequently reoccurring or contradictive themes are mentioned as headings in the result section. The relevant findings were first discussed with another researcher (AB) to acquire correct interpretations. Secondly, it was discussed which quotes could be used and which quotes contained enough rich substantiating content.

### Ethical considerations

The study was approved according to methodological and ethical standards and regulations of the University of Humanistic Studies. Informed consent was obtained from all of the participants in collecting and analysing the data anonymously from the interviews and the observations. Participation to this study was voluntarily and all participants could withdraw at any time. The confidentiality of all data is maintained in accordance with the General Data Protection Regulation.

## Results

### The term ‘values’

The respondents express difficulties in defining the concept of “values”. Most of the respondents talk about their own values in their work or indicated that they do not know how to define the term “values”. Nevertheless, some provide an explanation of the term “values”. They suggest that values differ between persons, that values are subjective constructs and that values can conflict with other values. They indicate that values have a purposeful meaning: values indicate what someone finds important and what someone can strive for. In addition, if an individual can live according to their own values, then this provides happiness and quality of life. None of the occupational physicians mentions in one or another way the guidelines of the NVAB about values. This can be seen in the following quotation:

Values determine whether you can be happy. Happiness within your life and what you have in your life. The possibility to stay close to yourself. If you know yourself well and know what you need, you can also look for relaxation in the present and enjoy […] the quality of life. (Transcript 9)

### Impeding and fostering factors

#### A relationship of trust

Almost all of the respondents mention trust, or having a relationship of trust with an employee, as a facilitating aspect for discussing values. The participants emphasize that a relationship of trust, where employees can experience freedom of speech, should be created, for example:

It happens regularly that someone had a passion somewhere else at a different employer or another job. Is it the right path to stay with their current employer? So, you must gain trust with the people you have to guide. (Transcript 5)

#### Time

The respondents indicate that they have little time to pay attention to values because their consultations only last 30–45 minutes (including writing up a report). In addition, they guide 15 employees in one day, so there is hardly time for reflection. Meanwhile, when an occupational physician has follow-up conversations with the employee, this benefits a relationship of trust and might therefore also be an advantage to talk about values, for example:

Well, it’s not possible with every employee. We also have people with a short procedure. Then I cannot make that deepening process, you need several appointments for that. It will certainly happen to employees who have a conflict at work or employees who are absent for four months. (Transcript 9)

#### Purely physical

According to the respondents, not all health problems are always suitable for a conversation about values. For example, when an employee has a purely physical complaint, such as a broken wrist, there is mostly no interest in a discussion about values. Meanwhile, psychological complaints such as burnout or depression can be a good starting point to talk about the employee’s values, for example:

It is certainly the case that people who suffer from burn out must work under great psychological pressure, it is nice to hear where they get energy from. (Transcript 5)

#### Educational attainment

Occupational physicians state that employees with less education show less concern for their values when compared with higher-educated (university degree) employees. According to some respondents lower-educated employees mainly work to earn money, while the higher-educated employees also work towards a career or to contribute to society. Therefore, some respondents think that it is less profitable to look at the motivation and values of the lower-educated, for example:

This also differs per sector, age, and education level. The simpler the form of work, the less there is a need to assign values to work. (Transcript 2)

#### Referring to a psychologist or social worker

If an employee is a long-term absentee (i.e., 6 weeks or longer) from work, then occupational physicians will usually refer them to a social worker or a psychologist to investigate further complaints, such as a burnout. The social worker or psychologist can investigate the motivation and values of the employee, and has time to go more in-depth, for example:

Awareness goes slowly and sometimes the psychologist has to support in this situation. (Transcript 9)

#### Assessor

Some employees have a negative image of the job of an occupational physician. In particular, many employees identify occupational physicians as an assessor, where an employee has to justify or defend themselves against the decision of an occupational physician. Therefore, some employees experience resistance in visiting an occupational physician and are not interested in a conversation about values or personal matters, for example:

Especially about occupational physicians people have a different image. They think: “You are going to form a judgement and I have to go back to work because what I say is not correct”. (Transcript 8)

#### Conflicting values

Conflicting values between the occupational physician and the employee may impede any discussion of values with an employee. Different values that originate from differences in religion, culture, or belief can cause disagreements, and thus do damage to the relationship of trust, for example:

Sometimes you get the well-known story: “Yes, I had a very nice party in the weekend and on Monday I call in sick”. […] Sometimes you do get those people to assess […] I do have some difficulty with that. (Transcript 4)

#### Different kinds of attention

There seems to be a difference to what extent occupational physicians pay attention to employee values. Some respondents find it important to pay attention to values because values can bring forward what an employee finds important in their work, which provides an opportunity to give appropriate advice. Paying attention to values can also help to make (life) decisions that grant an employee more happiness in life, as in the following example:

I think it is important to look into the values of employees. When I look into the values of the employees, I always look into: what is the value of your health, how important is it to you and what is the value of your work? (Transcript 2)

However, most respondents find that it is less important to pay attention to employee values. They do recognize that values play a role in their work, but mainly pay implicit or intuitive attention to this. They indicate that values quickly shift to the subconscious. The observations also show that values are mainly lived implicitly and it remains unclear whether the occupational physician is aware that certain values pass by during the conversation. In addition, the respondents do not use the word “values” much in a literal sense but talk instead about the employee’s “motivation” and “energy sources”. Questions like: “Do you enjoy your work” or “what motivates you” seem to be asked regularly, for example:

I’ve never used the word “values” in my room […] I do want to know: how do you feel in life? What is important to you? What are your hobbies? What does your social situation look like? Where are you from? I want to know things like that in order to understand why someone does as he does. (Transcript 8)

#### Importance of objectivity

Some participants presume that they are expected to provide objective medical advice from a neutral standpoint, which is not in favour of either the employee or the employer. For some participants, this seems to have implications for their attention to values. In particular, they consider values as subjective, which conflicts in giving objective advice. Therefore, some respondents leave out the attention to employee values. Moreover, when a situation becomes too complicated and too many values are playing a role between different stakeholders (e.g., in a conflict), some respondents return to a purely medical assessment of an employee and disregard any values, which can be seen in the following quotation:

Values are subjective. And you must make your judgement basedon objectivity. […] So don’t go too deep into those values. When it’s getting complicated you often take a step back and say: “You’re here with an occupational physician and I’m making an assessment based on medical factors.” […] Instruments have been provided to not to get submersed the quagmire of values. (Transcript 1)

### The employees’ different values

The respondents hardly name any forms of intrinsic motivation, such as “job interest”. Instead, they do say that they investigate whether an employee is motivated and if employees like their work. Hereby, it seems that occupational physicians are implicitly concerned with the value of “job interest”, especially when the employee seems to show a lack of enjoyment in their work, for example:

If you can see that someone is not happy with his work or private life, I will discuss it […] One thing I always suggest to employees is: ‘what do you really want? If it turns out that someone wants to lie down under a palm tree in Hawaii, but […] is working at a local supermarket then something goes wrong. I will ask how can you get there? And we talk about meaning and purpose in life. (Transcript 4)

Occupational physicians often mention extrinsic values, such as earning money, as a motivation for employees to work because it provides the possibility to cover basic needs and expenses. When most respondents talk about “earning money”, they indicate that other values also play an important role, which can be seen in the following example:

It is definitely more than just making money and meeting financial obligations. I notice that challenges, motivation, social contacts … There are a lot of factors that make work important for people. (Transcript 4)

In contrast, a number of participants state that a lot of employees purely work to earn money. Thereby, occupational physicians label certain work activities (e.g., working in a factory) as “simple” or “less meaningful”, where only “earning money” and “experiencing job security” remain as work values, for example:

But I think that if you come from secondary vocational education and you end up behind the assembly line in a chicken slaughterhouse, then I don’t think there is any other motivation than: “I do this dirty work because I’m making money”. (Transcript 6)

Occupational physicians state that prestige-oriented values are an important motivation for many employees to work. The work value “prestige” was also mentioned during one of the observations. According to the respondents, obsessive pursuit of prestige can lead to burnout complaints, which they notice increasingly:

In the past […] you had the trainer Rinus Michels and he said: “football is war”. Yes, and you will find out that labour is war as well. It is often like being on top of the heap, where all kinds of monkeys interact with each other. (Transcript 1)

The participants state that they regularly encounter employees who find social values such as “contributing to society” important. According to the participants, contributing to society can offer a certain degree of meaning and satisfaction, it brings forward a feeling of significance, for example:

That the light is on again because of you or that a train is in time because of you. That you really added and accomplished something […] Yes that you as a human have caused something good, something useful. (Transcript 8)

Finally, some of the occupational physicians report that the employees can find it important to have “contact with colleagues”. In particular, they notice during the COVID-19 pandemic that a lot of the employees miss contact with their colleagues because they worked from home.

## Discussion

The first goal of the present study was to understand the occupational physician’s comprehension of the term “values” and whether they adhere to the NVAB guidelines. None of the respondents mentioned or referred to the NVAB guidelines on values in their conversations. In addition, the respondents found it difficult to define the term “values”. The respondents who were able to provide a definition stated that values differ per individual, are subjective constructs, and can bring forward a feeling of happiness and quality of life; as also mentioned by Schwartz (2012), and Alma and Smaling (2010). The view that values are lived *implicitly* as stated by Schwartz (2012) was also mentioned by some respondents. Therefore, it is not surprising that occupational physicians predominantly pay implicit attention to values. Nevertheless, a minority stated that paying attention to the employee’s values is important and can benefit the outcome. According to these respondents, paying explicit attention to intrinsic values could contribute to the overall quality of collaboration and decision making, and align with the employee’s values and sense of meaning.

The second goal was to determine which factors foster or impede the employee’s attention to values. Because values are subjective constructs (Schwartz, 2012), some respondents had difficulties addressing values because they report that they are expected to provide an objective medical assessment. Moreover, some respondents hold that it is not necessary to gain insight into the employee’s values in order to accomplish an objective assessment. Additionally, the importance of trust and safety were mentioned. Chandra et al. (2018) specify that a relationship of trust and safety between a physician and patient is important and contributes to the quality and effectiveness of care and patient satisfaction. The respondents also mentioned time as an impeding factor. Lack of time is a common phenomenon among many physicians and can influence the satisfaction about the consultations, both for the employee and the occupational physician (Shirley & Sanders, 2013). Additionally, the negative image that some employees have of occupational physicians can influence the openness to discuss values. Moreover, employees are not free to choose their occupational physician, which negatively affects the relationship between the employee and the occupational physician (Plomp, 1992). Finally, conflicting values and experiencing different values (e.g., another cultural background or religious conviction) were mentioned as impeding factors.

The third goal was to determine how occupational physicians pay attention to employee values and which work values they notice in their conversations with the employees. The values that were mentioned by the occupational physicians are largely in line with the literature (Elizur et al., 1991; Ros et al., 1999), and only differ in the weight of importance. The respondents mainly discussed forms of extrinsic, social, and prestige-oriented values, but hardly discussed intrinsic values. In contrast, Elizur et al. (1991) indicated that intrinsic values receive a relatively high ranking among 515 Dutch participants. Moreover, intrinsic values correlate with greater life-satisfaction and less depressive symptoms than extrinsic values (Ling et al., 2016; Rigby & Ryan, 2018). Different values may come into play with employees who are not able to work when compared with healthy employees.

The question remains whether the guidelines about work values from the NVAB are intended as an aspiration or as directives to be implemented in practice. Plomp et al. (2012) state that many occupational physicians have difficulty complying with the NVAB guidelines. Although our respondents were familiar with the existence of NVAB guidelines in general, none of them mentioned the values guidelines. Occupational physicians are not trained in recognizing values and say that they have little time to pay attention to values. With respect to the assumed lack of time, one may wonder whether sufficient time is a decisive factor. One might imagine that discussing values can also form an essential subject that may enhance effective communication, such as a quick sense of being recognized or understood. Effective physician-patient communication is important for improving safety and overall satisfaction (Burgener, 2017; Shirley & Sanders, 2013). Therefore, more research is necessary to evaluate how the attention to values can serve effective communication.

### Recommendations (for occupational physicians and NVAB)

The present study shows that occupational physicians pay almost no explicit attention to the employee’s values. And if they do, it is mostly implicit, unconscious, or intuitive. It seems that the respondents did not feel the urge to follow the NVAB guidelines, even though some respondents agreed that paying attention to employee values could strengthen the collaboration. Hence, we recommend to train occupational physicians in discussing and recognizing values, to understand and practice values from the heart just as Guillemin and Nicholas (2022) propose. Through recognizing and acknowledging values, occupational physicians may be able to provide more appropriate advice in alignment with both the wishes and desires of the employees and the NVAB and increase employee well-being and health (Guillemin & Nicholas, 2022). Employees who live their lives in congruence with their intrinsic values and those of the organization may experience a higher sense of meaningfulness (Rosso et al., 2010; Sagiv & Schwartz, 2000; van Tuin et al., 2021). Another recommendation may be to introduce a spiritual counsellor to strengthen the awareness of values and meaning-making within the occupational health practice. Such a counsellor can also help to answer life questions such as: Who am I? Why do I work and live? What is a good (work) life? Currently, spiritual counsellors have not yet been engaged within the practice of occupational health services. Finally, the NVAB could consider strengthening the awareness of their guidelines under occupational physicians to promote the attention for employee values. Also, the NVAB could further investigate how values can play a role in the practice of the occupational physician, and how occupational physicians can pay attention to employee values. There is more data needed on how to deal and use values in the practice of the occupational physician.

### Limitations and further research

The first limitation of the present study was the relatively small, non-random sample. The occupational physicians who responded and agreed to participate may have had a positive attitude towards the subject of values, which may have influenced the results. Therefore, follow-up research should be conducted among a larger number of occupational physicians. The second limitation is to involve the employees by examining how they experience their work, whether and how they would prefer consultation with an occupational physician and if a conversation about values would be beneficial. This can be done through narrative research. The third limitation was the difference in conceptual terminology of the respondents and the interviewer. The interviewer used terms such as “values” and ‘meaning or “purpose”, whereas the respondents spoke more in terms of “energy sources” or “motivation”. Therefore, it remains unclear to what extent the researcher and the respondents actually meant the same thing. Values, motivation, and meaning are complex concepts that do not have a fixed definition within the literature. It is difficult to determine how and in what way values play a role in practice. Finally, within the context of occupational medicine, the present study does not provide immediate support for either the sustainable employability model or the capability approach. However, it identifies the challenge and task to translate the awareness of work-values into recognizable terms, both for employees and practitioners. The metaphor of “finding energy”, as mentioned by the respondents, may serve as an example. Translating work-values into accessible metaphoric cues could be subject for further inquiry.

## Conclusion

There is considerable a variety in the ways and degrees of how occupational physicians pay attention to the values of the employees. Some of the respondents focus on objective assessment and mostly chose to disregard employee values. In contrast, other respondents found it important to pay attention to values because they feel it helps them to give better advice to the employee. However, it remains unclear to which extent they do this in practice. The present study shows that the NVAB guidelines mostly remain unexecuted and underexposed in practice. We believe that heeding employee values in the practice of occupational health will contribute to the overall guidance of the occupational physician and supports employee health. However, further research is required into how heeding to employee values contributes to the overall quality of guidance and consultation of occupational physicians.

## Data Availability

The datasets (interviews and observations) are stored on servers from the University for Humanistic Studies. The dataset is not open or shareable for other researchers.

## Appendix 1: Interview guide

*Introduction:*

- Short Introduction of the research and the interviewer’s background.

- Request permission to process data through informed consent.

- Request permission for recording.

- Indicate time limit (60 min/75 min)

- Indicate freedom of speech (giving the opportunity not to answer questions if they don’t want to).

*Brief Introduction of the participant:*

- Introduction

- Background

- Opinion on the job of an occupational physician

*Starting questions:*

• What do you comprehend by the term ‘values?

• To what extent are you concerned with employee values?

• To what extent do you think attention to employee values is important?

• How do you pay attention to values in conversations with employees?

• What are obstacles talking about values? What is not helpful during conversations?

• What factors encourage talking about values? What is helpful during the conversations?

• Do you think employees benefit if you pay attention to their values? Why/why not? Which benefits?

• What values do you encounter in your own work?

• What values do you encounter among employees? And which values seem to provide meaning in their lives?

• Other comments/questions?

### Appendix 2:

24 work values according to Elizur, Borg, Hunt & Beck (1991) (ranked by importance according to Dutch respondents) and separated into four categories: intrinsic, extrinsic, social and prestige (Ros et al., 1999) as showed in the chart.

**Table:**

1. Job interest, to do work which is interesting to you	Intrinsic
2. Achievement in work	Prestige
3. Co-workers, fellow workers who are pleasant and agreeable	Social
4. Opportunity for personal growth	Intrinsic
5. Independence in work	Prestige
6. Advancement, changes for promotion	Prestige
7. Influence in work	Prestige
8. Use of ability and knowledge in your work	Intrinsic
9. Supervisor, a fair and considerate boss	
10. Opportunity to meet people and interact with them	Social
11. Responsibility	Intrinsic
12. Recognition for doing a good job	Prestige
13. Meaningful work	Intrinsic
14. Job security, permanent job	Extrinsic
15. Esteem	Prestige
16. Feedback concerning the results of your work	
17. Influence in the organization	Prestige
18. Pay, the amount of money you receive	Extrinsic
19. Benefits, vacation, sick leave, pension, insurance, etc	Extrinsic
20. Work conditions, comfortable and clean	
21. Convenient hours of work	
22. Company, to be employed by a company for which you are proud to work	Prestige
23. Contribution to society	Social
24. Job status	Prestige

### Appendix 3:

What does a Dutch occupational physician do:

**Table:**

The main goal of an occupational physician is to determine if an employee is (temporarily) not able to work. An employer can never decide this. Employers must report their ill employee to an occupational physician. Within 6 weeks, the occupational physician conducts a problem analysis, followed within a plan of action.
Occupational physicians guide the reintegration process after prolonged sickness and advise the employee and the employer on resuming work. Occupational physicians maintain confidentiality towards the employer. The occupational physician does not treat employers, but refers to other healthcare professionals if needed.
Occupational physicians advise employers on preventing absenteeism, aiming to keep employees healthy and motivated. Also, they periodically medically examine healthy employees.
Occupational physicians are registered healthcare professionals under the “Beroepen in de Individuele Gezondheidszorg” (Rijksoverheid, 2024) also known as B.I.G. registration (which protects patients against incompetent and negligent actions), possess extensive knowledge of psychological and physical conditions and healthcare.
An occupational physician is a neutral advisor and recognizes the interests of both employer and employee, and knows the rights and obligations of both parties.
